# Editorial: A Conversation With the Brain: Can We Speak Its Language?

**DOI:** 10.3389/fnins.2020.00794

**Published:** 2020-08-14

**Authors:** Alejandro Barriga-Rivera, Tianruo Guo, Yuki Hayashida, Gregg J. Suaning

**Affiliations:** ^1^School of Biomedical Engineering, The University of Sydney, Sydney, NSW, Australia; ^2^Department of Applied Physics III, University of Seville, Seville, Spain; ^3^Graduate School of Biomedical Engineering, UNSW Sydney, Sydney, NSW, Australia; ^4^Graduate School of Engineering, Osaka University, Osaka, Japan; ^5^Graduate School of Engineering, Mie University, Mie, Japan

**Keywords:** bionics, electrostimulation, nervous system, prosthesis, cochlear, modeling, electrode, neural interface

Hearing, sight, touch, or learning, all happens in the brain. The different organs in charge of sensing the environment send complex neural messages to the brain to inform about the surrounding world. Likewise, the brain sends different instructions to the organs to elicit a response such as a muscle contraction. Furthermore, the brain is also responsible for the different mental actions such as cognition or the generation of emotions. However, disease or trauma can alter the said neural communications causing blindness, deafness, paralysis, or mental illness among others. Luckily, a family of therapies based on the delivery of electric charge exists or are being investigated to treat some of these health conditions. An example of a successful treatment to restore audition is the cochlear implant (Zeng et al., 2008). Visual and motor prostheses provide hope to the blind and the paralyzed respectively. All of these medical devices share one common challenge: the replication of neural codes. This ambitious goal requires (1) the development of better ways to “listen” to the neurons by means of improved electrode-tissue interfaces and signal processing algorithms, (2) devising stimulation strategies able to mimic physiological responses, and (3) enhancing or restoring brain computational capabilities (Barriga-Rivera et al., 2017a).

This Research Topic includes a total of 11 contributions from more than 40 world leading experts and upcoming researchers, and provides a state-of-the-art view on some of the key questions related to our ability to maintaining a conversation with the brain to treat disease. Ranging from highly sophisticated computational models to novel brain tissue alternatives, the works presented here suggest new strategies to overcome some of the difficulties engineers and scientists are facing.

## Interpreting the Neurons

The quality of the conversation between the brain and devices highly depends on the goodness of the connection established with the neurons. On the one hand, computational models have demonstrated an enormous applicability in predicting the efficacy of the said connection and, in particular, how the electric fields generated by implanted electrodes can activate different neurons. For example, Bai et al. used micro-CT scans to reconstruct the detailed three-dimensional anatomy of the human cochlea which was then incorporated into finite element computational models of neural excitability. Along these same lines, a different modeling study (Bachmaier et al.) reported on the potential weaknesses of the mostly-used computational models of auditory nerve fibers. With these modeling studies, we discovered that limited biological features in the simulated nervous system, particularly missing anatomical microstructures and biophysical details, might cause inaccurate or even misleading information. On the other hand, sophisticated signal processing algorithms can assist in choosing the optimal message to be delivered to the brain. On this topic, a study on noise suppression in bionic hearing reminded us that signal processing can articulate superior performance in delivering information to the brain (Zhou et al.). However, an unmet need for improved electrode-tissue interfaces remains. A study by Gilmour et al. describes a new tool for testing of brain-electrode interfaces: “An improved *in vitro* model of cortical tissue.” As it integrates different cell types (astrocytes, microglia, oligodendrocytes, and neurons), this cost-effective approach can be used for large-scale preclinical evaluation of new-generation devices.

## Eliciting Meaningful Neural Activity

One of the key limitations in the field of neural electrostimulation relates to its poor ability to replicate physiological neural patterns (Borst and Theunissen, 1999). The development of many neural prostheses has reached an impasse where the level of artificially elicited function does not warrant implanting these devices in more than an experimental-scale cohort of patients. Over the last decade, novel stimulation methods have been developed to directly address the challenge of being able to restore some of the natural processes that occur with normal function through the control of critical neural pathways. For example, high-frequency stimulation (Guo et al., 2017; Muralidharan et al., 2020) or field shaping techniques (Cicione et al., 2012; Barriga-Rivera et al., 2017a,b) have been investigated to improve artificial vision. State-of-the-art stimulation strategies in the field of bionic vision have been updated in this topic (Fernandez et al.; Tong et al.). In addition, Saeedi's and Hemmert's research work (Saeedi and Hemmert) shows new insights on how neural information elicited by multi-pulse electrical stimulation integrates within the auditory brainstem in 12 cochlear implant recipients. Other researchers (Vickery et al.; Yap et al.) provided an update on the current status of transcutaneous nerve stimulation, whereas Loulit and Potas proposed the dorsal column nuclei as a target for somatosensory restoration.

While there are many studies in this special issue devoted to expanding our understanding of how artificial electrical stimulation interact with neurons with the hope of improving the quality of the artificially elicited neural activity, most of the proposed stimulation methods will require supporting of improved material, manufacturing and packaging techniques to eventually reach the clinic (Rivnay et al., 2017; Benfenati and Lanzani, 2018; Levi et al., 2018).

## Enhancing Brain Computational Power

Paraphrasing the first words of this editorial, everything occurs in the brain. It is therefore the ultimate target of nearly all afferent neuromodulation applications. While improving neural interfaces and signal processing techniques is essential to delivering meaningful neural messages, the brain has the last word in the interpretation of those messages. Fernandez and colleagues (Fernandez et al.) pointed to the potential the brains of the blind have to adapt to the re-introduction of a visual input. The authors remarked on the importance of devising rehabilitation strategies to potentiate the brain capacity of coping with artificially encoded neural messages, a practice that could plausibly bring the performance of neural prostheses to a superior level (Beyeler et al., 2017).

## Final Remarks

The brain is an extraordinarily complex organ that integrates over 100 trillion connections from nearly 100 billion neurons. In this topic, Buskila et al. remind us of the importance of other brain cells such as the astrocytes in the generation of brain states, a phenomenon known as lateral astrocytic synaptic regulation. In other words, the brain works as a perfectly coordinated orchestra with many instruments of different kinds. When disease or accidents alter the score or the composition of the orchestra, a different tune is played. To restore or even mimic the lost function, the many neurostimulation strategies under development and investigation require a highly multi-disciplinary approach to be able to face the general problem from different viewpoints. Technological advancement can only be accelerated by establishing stronger collaborations between clinicians, neuroscientists and biomedical engineers.

## Author Contributions

All authors listed have made a substantial, direct and intellectual contribution to the work, and approved it for publication.

## Conflict of Interest

The authors declare that the research was conducted in the absence of any commercial or financial relationships that could be construed as a potential conflict of interest.
